# Characterization of the complete mitochondrial genome of the nematophagous fungus *Purpureocillium lavendulum*

**DOI:** 10.1080/23802359.2020.1846000

**Published:** 2021-01-05

**Authors:** Lian-Ming Liang, Ying Zhang, Jianping Xu, Ke-Qin Zhang, Yan-Ru Cao

**Affiliations:** a State Key Laboratory for Conservation and Utilization of Bio-Resources in Yunnan and The Key Laboratory for Southwest Microbial Diversity of the Ministry of Education, Yunnan University, Kunming, China; b Department of Biology, McMaster University, Hamilton, Canada; c Key Laboratory of Special Biological Resource Development and Utilization of Universities in Yunnan province, College of Agriculture and Life Sciences, Kunming University, Kunming, China

**Keywords:** Nematophagous fungus, *Purpureocillium lavendulum*

## Abstract

The complete mitochondrial genome of *Purpureocillium lavendulum* was characterized in this study. This mitogenome is a closed circular molecule of 23,567 bp in length with a GC content of 28.46%, including 15 protein-coding genes, 25 transfer RNA genes, 2 ribosomal RNA genes. Phylogenetic analyses based on sequences at the 14 concatenated mitochondrial protein-coding genes showed that *P. lavendulum* was closely related to *Hirsutella minnesotensis*.

Plant-parasitic nematodes (PPNs) cause severe damage to agricultural crops worldwide (Liang et al. 2019). The nematophagous fungus *Purpureocillium lavendulum* (Pezizomycotina, Sordariomycetes, Hypocreales, Ophiocordycipitaceae) was previously mis-identified as *Paecilomyces lilacinus*, a commonly used fungus to control agricultural nematodes, such as the root-knot nematode *Meloidogyne incognita* (Jatala et al. 1980). Recently, the mitochondrial genomes have been reported from a number of nematophagous fungi species (Deng and Yu 2019; Fang et al. 2019; Li and Yu 2019; Wang et al. 2019; Zhang and Yu 2019), which provides novel insights into the evolution of nematophagous fungi and facilitate further investigations of this ecologically and agriculturally important group of fungi (Zhang et al. 2020). The genus *Purpureocillium* was proposed in 2011 (Luangsa-Ard et al. 2011) and *P. lavendulum* was proposed as a new species in 2013 (Perdomo et al. 2013). We have created a high efficient genetic manipulation system for this fungus, thus facilitate the characterization of biological functions of nematode infection-related genes (Liu et al. 2019). Based on the system, key regulators in conidiation procedure in *P. lavendulum* have been characterized (Chen et al. 2020). However, the genetic information of its mitochondria has not been analyzed. In this study, we report the complete genome of *P. lavendulum* and investigate its phylogenetic relationship with other ascomycetous species. The annotated genomic sequence was submitted to GenBank under accession number MW019427.

The mitogenome was extracted from the whole genome assembly of *P. lavendulum* which we have sequenced using HiSeq4000 PE150 and PacBio RSII (unpublished data). The whole mitochondrial genome was annotated automatically using the Mitos2 tool (http://mitos2.bioinf.uni-leipzig.de/index.py) based on the Reference Ref 63 Fungi and Yeast 3 code (Bernt et al. 2013). tRNAs were annotated using tRNAscan-SE (Schattner et al. 2005). rRNAs were annotated using hmmer3 based on the database Rfam14 (Johnson et al. 2010).

The complete mitogenome of *P. lavendulum* is a closed circular molecule of 23,567 bp in length with a GC content of 28.46%, which contains 42 genes, including 15 mitochondrial protein-coding genes (PCGs), 25 tRNA genes (tRNA-Trp gene lacked), and 2 rRNA genes. Protein-encoding genes include three ATP synthase subunits (atp6, atp8, and atp9), three cytochrome oxidase subunits (cox1, cox2, and cox3), one apocytochrome b (cob), seven NADH dehydrogenase subunits (nad1, nad2, nad3, nad4, nad4L, nad5, and nad6) and one ribosomal protein (rps3). No introns were existed in all the predicted genes. All tRNA genes are encoded on the sense strand. All PCGs initiated with ATG as the start codon.

Phylogenetic analysis of 14 mitochondrial proteins of *P. lavendulum* and other 18 species in Ascomycota was performed by Bayesian inference (BI). As shown in Figure 1, *P. lavendulum* and *Hirsutella minnesotensis* clustered together and related to *Cordyceps brongniartii*, *Fusarium moniliformis*, *Colletotrichum acutatum* and *Sclerotinia borealis*, which are all members from Sordariomycetes.

**Figure 1.**
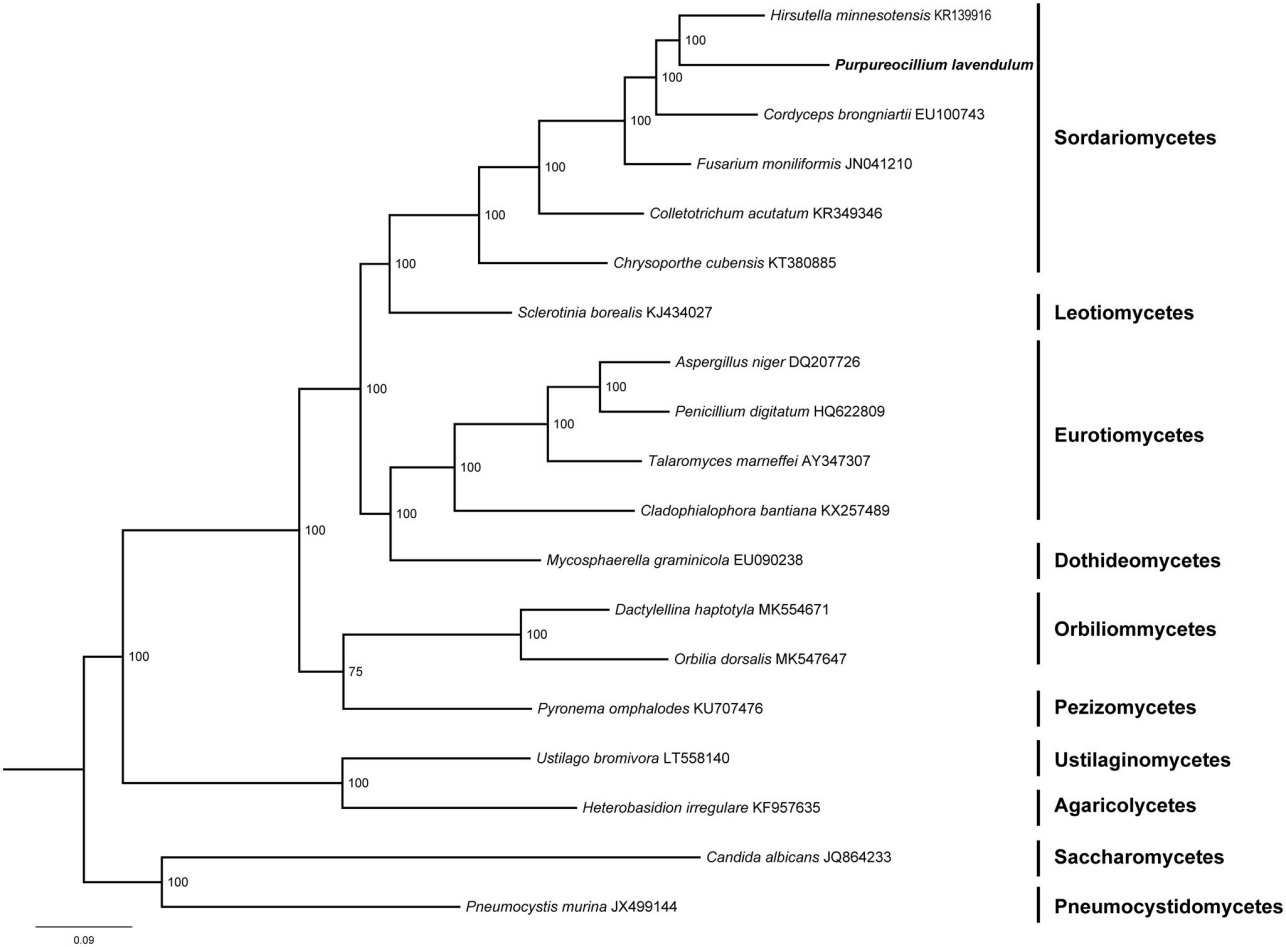
Phylogenetic relationships among 19 Ascomycota fungi inferred based on the concatenated amino acid sequences of 14 mitochondrial protein-coding genes (atp6, atp8, atp9, cox1, cox2, cox3, cob, nad1, nad2, nad3, nad4, nad4L, nad5 and nad6). The tree was generated using MrBayes. Values along branches represent statistical support based on 1000 randomizations.

## Data Availability

The data that support the findings of this study are openly available in the GenBank (accession no. MW019427) at https://www.ncbi.nlm.nih.gov/genbank/.
